# Micro-URS Experience in the Treatment of Distal Ureteral Stones in Preschool-Aged Children

**DOI:** 10.3390/jcm14072500

**Published:** 2025-04-06

**Authors:** Mehmet Mazhar Utangac

**Affiliations:** Department of Urology, Faculty of Medicine, Dicle University, 21280 Diyarbakır, Turkey; drmazhar21@hotmail.com; Tel.: +90-533-816-23-88; Fax: +90-412-248-80-01

**Keywords:** micro-ureteroscopy, distal ureteral calculi, children

## Abstract

**Objective:** The incidence of urolithiasis in the paediatric population is rising, leading to a progressive shift towards minimally invasive management strategies. This study evaluated the efficacy and safety of using micro-ureteroscopy (micro-URS) to treat distal ureteral stones in preschool-aged paediatric patients. **Methods:** A retrospective analysis was conducted on 57 children (aged 6–72 months), all of whom had undergone micro-URS treatment for distal ureteral stones between September 2022 and April 2024. Patient demographics, along with perioperative and postoperative outcomes, were assessed. Stone fragmentation was achieved using a 4.85 Fr micro-ureteroscope and a 200 μm Ho:YAG laser fibre. Postoperative complications were graded according to the Clavien–Dindo classification system, and stone-free status was confirmed for each patient at their one-month follow-up appointment. **Results:** The mean patient age was 44.2 months, and the median stone size was 9.4 mm (range: 6–24 mm). Stone-free status was confirmed in all patients at their one-month follow-up appointment. In 22.8% of cases, reintervention was required to address minor complications, including haematuria (*n* = 6), urinary tract infections (*n* = 4), and stone migration (*n* = 3). No major intraoperative complications were observed. A total of 41 patients (71.9%) required a double-J stent to treat intraoperative oedema or stone impaction. The mean operative time was 28.6 min, and the mean hospitalisation duration was 19.7 h. **Conclusions:** Micro-URS achieved a 100% stone-free rate with minimal complications, establishing it as a safe and highly effective option for treating distal ureteral stones in preschool-aged children. These findings show that micro-URS offers advantages over Shock Wave Lithotripsy (SWL) in paediatric urolithiasis management, supporting it as a first-line treatment modality. Further prospective, randomised studies are needed to validate these results.

## 1. Introduction

The prevalence of urolithiasis in the paediatric age group is increasing [1]. Consequently, diagnoses are being made in a greater number of children of younger ages [1]. The recurrence rate of stone disease in the paediatric age group is higher compared to adults [2]. This high recurrence risk necessitates that the chosen treatment method be as minimally invasive as possible.

Shock Wave Lithotripsy (SWL) and ureteroscopy (URS) are the most commonly used methods for treating ureteral stones [3,4]. Various factors support URS as the superior treatment option, including the necessary use of anaesthesia in paediatric SWL treatment; the inability to fragment a stone in a single session; the eventual need to proceed with URS treatment if SWL fails; and higher failure rates of SWL in focusing on distal ureteral stones [5]. The miniaturisation of ureteroscopes, the introduction of laser lithotripsy, and increased access to these technological advancements have enabled URS to gain superiority over SWL [6].

Given the relatively young age of the diagnosed patients in this population, surgical success relies heavily on the use of suitable equipment. Technological advancements in endourology have facilitated the miniaturisation of endoscopic instruments without compromising their effectiveness; these instruments have, in turn, increased the efficacy of treating urinary system stones in young children [1]. The literature reveals that the 4.8 Fr endoscope is the smallest endoscope used for managing renal stones via the percutaneous route, and it has also been employed in the treatment of bladder and distal ureteral stones [7,8]. This method—used in the management of cases with ureteral calculi—has been termed “micro-URS” [8,9,10]. The miniaturisation of endoscopic equipment in recent years has enabled the development of new minimally invasive surgical methods [11,12]. Using smaller calibre ureteroscopes can potentially reduce the risk of complications such as ureteral injury, bleeding, or tears as well as the need for postoperative double-J stent placement [1]. For this reason, micro-URS has become a more common approach to treating ureteral stones in paediatric patients.

The present study aimed to demonstrate the efficacy and safety of micro-URS in the management of distal ureteral stones in preschool-aged paediatric patients. To the best of our knowledge, this is the first reported series of micro-URS in preschool-aged paediatric patients in the literature.

## 2. Method

Approval for this study was received from the Dicle University Ethics Committee. Parental informed consent was given on behalf of the patients included in the study. A total of 57 children were retrospectively evaluated, all of whom had undergone micro-URS between September 2022 and April 2024, with the indication of distal ureteral calculi in one centre. The patients included in the study were not receiving any treatment for distal ureteral calculi. Symptomatic patients with severe hydronephrosis, vomiting, and >5 mm ureteral stones were included in the study. Asymptomatic patients and patients with <5 mm ureteral stones were excluded from the study. All the parents of my patients who will undergo surgery were provided with detailed information about all alternative treatment options. The ureter from the lower point of the sacroiliac joint to the entrance to the bladder is defined as the distal ureter. The data—including patient demographics, perioperative data, and postoperative data—were collected retrospectively.

Preoperatively, a routine physical examination and biochemical assessments were performed. In all cases, urine cultures were used to confirm the presence of urinary tract infections. Plain urinary system radiograms (KUB), ultrasonography, and/or low-dose computed tomography were used as imaging methods. Stone burden was expressed as the measurement of the longest diameter of the stone. In cases where multiple stones were present, the stone burden was expressed as the sum of the stone diameters.

### Micro-Ureteroscopy Instruments and Technique

**Figure 1:**
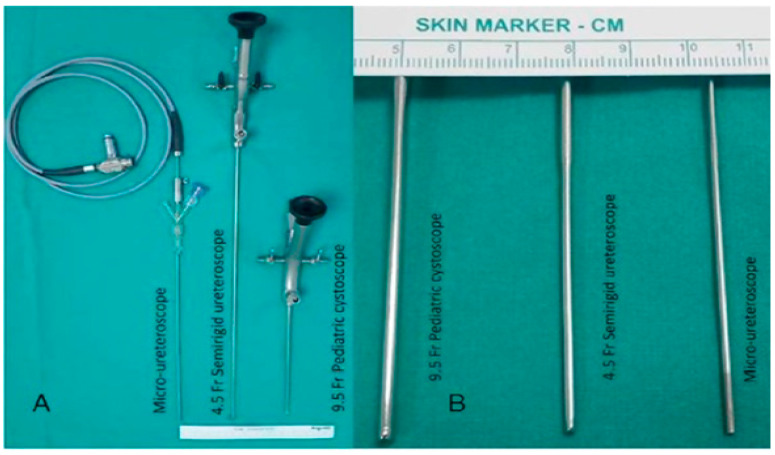
(**A**) Materials that can be used in the operation; (**B**) micro-ureteroscope diameter thinness.

A micro-ureteroscope is an instrument with a shaft size of 4.85 Fr and a lumen of 1.4 mm (Figure 1); an adaptor is attached to the proximal side of the instrument’s shaft, as well as a 0.9 mm optic that provides an image quality of 10,000 pixels (PolyDiagnost, Pfaffenhofen, Germany) (Figure 1). The optic is inserted into the lumen of the shaft through the second lumen of the adapter, which has a 3 mm diameter. The other lumens allow the insertion of the laser fibre (200 μm) and drainage of the irrigation fluid. During the procedure, irrigation was provided using a Y-TUR irrigation set with a pump handle.

**Figure 2:**
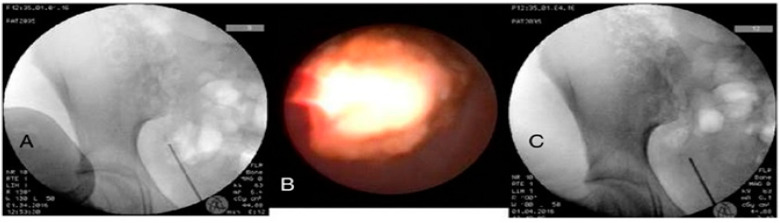
(**A**) Intraoperative scopy image taken before stone breakage; (**B**) endoscopic image taken before stone breakage; (**C**) intraoperative scopy image taken after stone breakage.

General anaesthesia was administered to each patient. Patients were then placed in the lithotomy position. Experienced surgeons proceeded to perform the procedure following the standard URS technique and using a telescope developed for micro-percutaneous nephrolithotomy [8]. During the procedure, a C-arm fluoroscopy device was set up and ready for use when necessary. In both male and female patients, the optic passed easily through the urethra to the bladder and ureteral orifice (Figure 2). Balloon dilation was not needed for access through the ureteral orifice. A guidewire was used to facilitate passage in patients with a tortuous ureter. In cases with blurred image quality, a manual irrigation pump system was used to obtain adequate image quality. Stone fragmentation was accomplished with a 200 μm Ho:YAG laser fibre using the dusting technique at a setting of 6 Hz and a power of 0.6 joules.

Double-J (DJ) stents were placed in the ureters with severe oedema as a result of stone impaction and injured ureters where stones were impacted. A guidewire was inserted through the shaft up to the upper urinary tract. Then, a 4.8 Fr DJ stent was inserted over the guidewire. Parenteral or oral analgesics (paracetamol 10 mg/kg per dose) were utilised for postoperative pain relief.

Using the KUB and US imaging modalities, each patient was evaluated on the morning of their first postoperative day and one month later. Postoperatively, the patient’s restlessness and nutritional status were evaluated. A total of 32 patients required oral analgesia. There was no need for oral analgesia during the long period until DJ stent removal. Two weeks after surgery, the DJ stent was removed endoscopically under sedation anaesthesia. Postoperative complications were graded using the Clavien–Dindo classification system.

## 3. Results

Patient demographics and perioperative data and outcomes are described in Table 1 and Table 2. Right (*n* = 31) and left (*n* = 26) ureteral stones were detected in the respective numbers of patients. The mean age of the patients was calculated as 44.2 months (range: 6–72). The main urinary tract infection symptoms presented were flank pain (24/57), haematuria (38/57), and fever (13/57). The median stone size was 9.4 mm (range: 6–24). Upper tract dilation and hydronephrosis were detected in all cases. In 41 of the 57 patients, a double-J stent was placed due to severe intraoperative oedema. The median operative time was 28.6 min (range: 21–65 min). In the postoperative period, stone migration was observed in three patients (Clavien grade III), mild haematuria occurred in six patients (Clavien grade II), and urinary tract infection (UTI) developed in four patients (Clavien grade I). No major intraoperative complications occurred in any of the cases. The mean hospitalisation time was 19.7 h (range: 12–32 h). The patients with migrated stones underwent repeat micro-URS within the first postoperative month, and complete stone-free status was achieved. Stone-free status was accomplished in all patients in the final assessment. During the first month control visit, no signs of residual fragments, hydronephrosis, or urinary tract infection were detected.

## 4. Discussion

The incidence of ureteral stones in paediatric patients is less frequent than that of adult patients [13]. It is critical to use surgical instruments appropriate for the ureteral diameter, as this lowers the risk of potential complications when treating ureteral stones [14]. This advantage makes minimally invasive approaches the gold standard of treatment [1]. Recently, the emergence of smaller instruments has provided new alternatives for the minimally invasive treatment of stone disease [1].

MicroPerc URS has reportedly been used as a semi-rigid ureteroscope for renal pelvic stones [1]. Some studies also indicate that MicroPerc URS has been used for ureteral stones [15]. In the paediatric population, the likelihood of spontaneous passage of ureteral stones is higher than in the adult population [15]. Therefore, conservative follow-up remains an alternative treatment option for certain ureteral stones. In the literature, stones smaller than 6 mm [16] and ≤3 mm [17] have been reported as likely to pass spontaneously. In another study, conservative management was recommended for stones ≤5 mm, while symptomatic stones larger than 5 mm were treated with URS [15]. In our study, consistent with the literature, URS was performed on symptomatic stones larger than 5 mm.

The primary goal of URS in the treatment of ureteral stones is to achieve a complete stone-free status. According to the literature, stone-free success rates range from 82% to 100% in paediatric patients treated with a Holmium:YAG laser, pneumatic lithotripters, or ultrasonic lithotripters [18]. In a study comparing pneumatic lithotripters and Holmium:YAG lasers, stone fragmentation success rates were found to be similar, although laser use was superior in terms of preventing stone migration and reducing operation time [19]. In our study, the stone-free rate in the first postoperative month was 100%, which was consistent with findings in the literature. We attribute our high success rate to the following factors: the location of the stones in the distal ureter, the high volume of stone cases handled by our centre, and the use of laser lithotripsy in all patients. Overall, our study supports the efficacy of micro-URS in treating preschool-aged paediatric patients, with a 100% stone-free rate and no residual fragments or hydronephrosis observed at the first postoperative month of follow-up.

The use of URS for ureteral stone treatment is associated with low morbidity and complication rates [15]. Minor complications during and after URS include mild mucosal injuries, stone migration to the kidney, minor haematuria, fever, and renal colic, while major complications include ureteral perforation, false passage formation, stone extravasation into the retroperitoneal area, fluid extravasation, ureteral rupture, sepsis, and even death [20]. Despite the use of low-calibre ureteroscopes, the literature reports complication rates ranging from 8% to 25% [14]. Consistent with the literature, the rate of minor complications in our study was 22.8%. Although the use of smaller calibre ureteroscopes is emphasised in the literature, failure and complication rates can still vary between 8% and 25% [14]. We preferred laser lithotripsy over pneumatic lithotripsy for stone fragmentation and did not encounter major complications such as ureteral perforation. Ureteral perforation is one of the most feared complications of URS. It usually occurs due to uncontrolled advancement of the ureteroscope or during lithotripsy [15]. The most common cause in children is the narrow ureteral orifice and ureteral diameter [14]. Thanks to the small calibre of the micro-URS, we did not experience any issues with accessing the ureter from the lower ureteral segment in any of our patients.

No consensus exists on the routine use of ureteral stents after ureteral stone surgery, but the generally accepted view is that stents may not be necessary in uncomplicated cases after proper stone fragmentation [21]. While some studies advocate for stentless URS to reduce postoperative discomfort and morbidity in children, the necessity of stent placement remains debatable, especially in paediatric patients with significant oedema [22]. A review of the literature reported that ureteral stent placement following URS is performed in 60–75% of cases [23]. Consistent with the literature, double-J catheters were used in 41 patients (71.9%) in our study due to ureteral oedema. In uncomplicated paediatric cases, we decided against placing DJ stents in order to prevent bladder spasms and avoid the additional anaesthesia required for stent removal.

The main limitations of our study include its retrospective design, single-centre experience, the lack of a comparison group, and the relatively small number of cases in this paediatric series. Prospective, randomised, and controlled studies are needed to promote the more widespread use of micro-URS in the treatment of ureteral stones in paediatric patients.

## 5. Conclusions

In conclusion, this study demonstrates that micro-URS is a safe and highly effective method to treat distal ureteral stones in preschool-aged children. Our findings contribute to research on the increasing use of micro-URS in the paediatric population and highlight its significant role as a first-line treatment.

## Figures and Tables

**Table 1:** Demographic data.

Laterality		
	Right	31 patients
	Left	26 patients
Average age	44.2 (6–72 months)	
Stone size average	9.4 mm (6–24 mm)	
Complaint		
	Side pain	24 patients
	Hematuria	38 patients
	Fever	13 patients

**Table 2:** Operational data of patients.

Operation time	28.6 (24–64 min)
Hospitalisation (hours)	19.7 (12–32 h)
DJ stent	22 (%38.5) patients
Complication	13 (%22.8) patients
	Mild hematuria, 6 patients, Clavien 1
	UTİ, 4 patients, Clavien 2
	Stone migration, 3 patients, Clavien 3
Stonefree 1st Month	57 (%100) patients

## Data Availability

The original contributions presented in this study are included in the article. Further inquiries can be directed to the corresponding author.

## References

[1] Parente A., Ortiz R., Fernández-Bautista B., Burgos L., Angulo J.M. (2021). Micro-Ureteroscopy as a Treatment of Renal Pelvis Lithiasis in Young Children. Front. Pediatr..

[2] Öner A., Demircin G., İpekçioğlu H., Bülbül M., Ecin N. (1997). Etiological and clinical patterns of urolithiasis in Turkish children. Eur. Urol..

[3] Preminger G.M., Tiselius H.-G., Assimos D.G., Alken P., Buck A.C., Gallucci M., Knoll T., Lingeman J.E., Nakada S.Y., Pearle M.S. (2007). 2007 Guideline for the management of ureteral calculi. Eur. Urol..

[4] Porfyris O.T., Cutress M.L., Tolley D.A. (2011). The use of extracorporeal shockwave lithotripsy for obstructing ureteric stones. Minerva Urol. Nefrol..

[5] Dwyer M.E., Krambeck A.E., Bergstralh E.J., Milliner D.S., Lieske J.C., Rule A.D. (2012). Temporal trends in incidence of kidney stones among children: A 25-year population-based study. J. Urol..

[6] Sani A., Beheshti R., Khalichi R., Taraghikhah M., Nourollahi E., Shafigh A., Pashazadeh F., Ghojazadeh M., Mostafaei H., Salehi-Pourmehr H. (2025). Urolithiasis management: An umbrella review on the efficacy and safety of extracorporeal shock wave lithotripsy (ESWL) versus the ureteroscopic approach. Urol. J..

[7] Desai M., Mishra S. (2012). ‘MicroPerc’ micro percutaneous nephrolithotomy: Evidence to practice. Curr. Opin. Urol..

[8] Caballero J., Galán J., Verges A., Amorós A., Garcia-Segui A. (2015). Micro-ureteroscopy: Initial experience in the endoscopic treatment of pelvic ureteral lithiasis. Actas Urol. Esp..

[9] Caballero-Romeu J.P., Budia-Alba A., Galan-Llopis J.A., Montoya-Lirola M.D., García-Tabar P.J., Galiano-Baena J.F., Albertos-Mira-Marcelí N., Gonzalvez-Piñera J. (2016). Micro-ureteroscopy in children: Two first cases. J. Endourol. Case Rep..

[10] Utanğaç M.M., Sancaktutar A.A., Tepeler A. (2017). Micro-ureteroscopy for the treatment of distal ureteral calculi in children. J. Pediatr. Surg..

[11] Caballero-Romeu J.-P., Galán-Llopis J.-A., Pérez-Fentes D., Budia-Alba A., Cepeda-Delgado M., Palmero-Marti J.-L., Cansino-Alcaide J.-R., Caballero-Pérez P., Ibarluzea-Gonzalez G. (2016). Assessment of the effectiveness, safety, and reproducibility of micro-ureteroscopy in the treatment of distal ureteral stones in women: A multicenter prospective study. J. Endourol..

[12] Caballero-Romeu J.-P., Galán-Llopis J.-A., Soria F., Morcillo-Martín E., Caballero-Pérez P., Garcia A., De La Cruz-Conty J.E., Romero-Maroto J. (2018). Micro-ureteroscopy vs. ureteroscopy: Effects of miniaturization on renal vascularization and intrapelvic pressure. World J. Urol..

[13] Raza A., Smith G., Moussa S., Tolley D. (2005). Ureteroscopy in the management of pediatric urinary tract calculi. J. Endourol..

[14] Uzun H., Akça N. (2018). Is the 4.5-F ureteroscope (Ultra-Thin) an alternative in the management of ureteric and renal pelvic stones?. Arab. J. Urol..

[15] Topaktas R., Aydin C., Altin S., Akkoc A., Aydın Z.B., Urkmez A. (2019). The efficacy of ultra-thin semi-rigid ureteroscopy with Holmium laser lithotripsy in pediatric ureteral stones: A single-center experience. Cureus.

[16] Dellabella M., Milanese G., Muzzonigro G. (2003). Efficacy of tamsulosin in the medical management of juxtavesical ureteral stones. J. Urol..

[17] Van Savage J.G., Palanca L.G., Andersen R.D., Rao G.S., Slaughenhoupt B.L. (2000). Treatment of distal ureteral stones in children: Similarities to the American Urological Association guidelines in adults. J. Urol..

[18] Lesani O.A., Palmer J.S. (2006). Retrograde proximal rigid ureteroscopy and pyeloscopy in prepubertal children: Safe and effective. J. Urol..

[19] Joshi H.N., Singh A.K., Koirala N.P., Karmacharya R.M. (2020). Outcome of uretero-renoscopic lithotripsy (URSL) with Holmium laser vs pneumatic lithotripter for lower ureteric stones: Experience from a university hospital in Nepal. Kathmandu Univ. Med. J..

[20] Dogan H.S., Onal B., Satar N., Aygun C., Piskin M., Tanriverdi O., Gurocak S., Gunay L.M., Burgu B., Ozden E. (2011). Factors affecting complication rates of ureteroscopic lithotripsy in children: Results of multi-institutional retrospective analysis by Pediatric Stone Disease Study Group of Turkish Pediatric Urology Society. J. Urol..

[21] Haleblian G., Kijvikai K., De la Rosette J., Preminger G. (2008). Ureteral stenting and urinary stone management: A systematic review. J. Urol..

[22] Abdelhafez M.F., Bedke J., Amend B., Elzayat E., Koehrmann K.U., Sievert K.D. (2013). Minimally invasive endourological management of pediatric urolithiasis: State of the art. J. Endourol..

[23] Galal E.M., El-Bab T.K.F., Abdelhamid A.M. (2013). Outcome of ureteroscopy for treatment of pediatric ureteral stones. J. Pediatr. Urol..

